# AI-driven drug discovery using transformer-based molecular representation learning

**DOI:** 10.3389/frai.2026.1807340

**Published:** 2026-07-01

**Authors:** V. Karthik, Mukunda Hosangadi, Sumedh Kudale, OmKumar Chandra Umakanthan

**Affiliations:** School of Computer Science and Engineering, Vellore Institute of Technology, Chennai, India

**Keywords:** Byte-Latent Transformer, cheminformatics, deep learning, drug discovery, molecular property prediction, SMILES representation

## Abstract

The vast majority of chemically plausible, drug-like molecules remain unexplored due to the combinatorial scale of chemical space and the limited throughput of experimental screening. This is complicated by the lack of data and the inability to extrapolate predictive models to chemotypes that are not represented well. We introduce a transformer-based molecular modeling framework for target-specific potency prediction, trained on curated BindingDB bioactivity data across Alzheimer‘s, diabetes, and cancer targets to deliver accurate pIC50 regression and binary activity classification. It uses curated bioactivity data from BindingDB to build target-specific datasets and uses a Byte Latent Transformer (BLT) that is trained directly on SMILES strings to predict changes in compound activity and potency based on quantitative structure–activity relationships. The transformer captures both syntactic and higher-level chemical features of SMILES representations and performs byte-level predictions using a latent model trained on the same molecular information. Potency predictions are executed inside an engine of chemically described molecular search engine, which executes stochasticity, SMILES-based amount mutations benefit by the envisaged activity, drug-like rules, and adaptive seeking heuristics to prevent local minima. Optimized candidate molecules and local optima are generated through guided SMILES mutations, preserving structural diversity around high-potency leads. The framework delivers highly accurate pIC50 regression (*R*^2^ 0.95–0.98) and binary activity classification across Alzheimer's, diabetes, and cancer targets, enabling robust virtual screening and lead prioritization for diverse biological targets.

## 1 Introduction

Even though there is a small number of compounds that have been characterized experimentally, the population of synthetically accessible, drug-like molecules is thought to be astronomical. Consequently, the vast majority of chemically feasible but experimentally untested molecular structures remain inaccessible to conventional high-throughput screening pipelines.

Computational techniques have now played a crucial role in exploring this space with specific attention to those techniques that can be used to predict molecular properties and optimize a compound before experimental confirmation. Classical structure-activity relation (QSAR) models of quantitative nature have been found useful, as well as more recent models based on descriptors (Rehman et al., 2025), but both methods are limited by their reliance on pre-defined molecular representations and manually engineered descriptors.

Sequence models that use transformers are an alternative paradigm to molecular representation learning, consisting of the view that chemical structures can be learned as structured sequences, not fixed descriptors. Specifically, Byte Latent Transformers (BLTs) process SMILES strings as raw byte sequences, enabling fine-grained modeling of molecular syntax while learning latent representations that capture higher-level chemical patterns. The methodology eliminates explicit fingerprint selection, is better tolerant to syntactic variation, and offers a flexible basis on which downstream prediction and optimization tasks can be performed.

Nevertheless, chemical space exploration (Ocana et al., 2025) must be paired with molecular optimization methods that propose synthetically accessible modifications while balancing exploration of novel scaffolds against refinement of known leads, all within drug-like chemical space constraints. Additionally, interpretability is crucial: chemists need to contextualize predicted potency improvements within broader structure–activity relationships, rather than relying solely on numerical pIC50 values.

To solve these problems, we introduce a combined computational system, which combines the predictive transformers of potency with adaptive optimization of molecules. BindingDB (Gilson et al., 2016) bioactivity data are also utilized to create target-specific datasets on which potency predictors based on BLT are trained. The model enables structure-guided lead optimization by predicting potency for SMILES variants obtained through targeted mutations, while applying drug-likeness filters and potency-based selection to identify improved candidates.

By combining transformer-based molecular representation learning (Fu and Chen, 2025) with optimization and visualization in a single, end-to-end workflow, this framework facilitates systematic exploration of underrepresented chemical space and supports data-driven decision-making in early-stage drug discovery.

## 2 Related work

Articles by Rehman et al. (2025), Ocana et al. (2025), Fu and Chen (2025), and Saini et al. (2025) provided comprehensive overviews of artificial intelligence transforming pharmaceutical R&D across the entire pipeline. Rehman et al. examined how machine learning and deep learning use large-scale biological datasets to improve disease diagnosis, target identification through AlphaFold, virtual screening via QSAR models, and ADMET profiling to reduce late-stage failures. Ocana et al. focused on the sphere of oncology, describing multi-omics analysis to detect oncogenic vulnerabilities, *de novo* design using generative models, and precision medicine using AI, as well as attempting to address the problem of data governance and multimodal integration. Fu, C. et al. proposed AI applications that optimize clinical trials and repurpose drugs based on their graph neural networks and transformer architectures, and identify AI-based target discovery as the most critical issues in data bias, low interpretability, and regulatory concerns. Saini et al. argued that the typical 10–15 year drug development timeline can be shortened through machine learning-based SAR modeling, in silico clinical trial simulation, and manufacturing optimization—provided human oversight and standardized validation frameworks address model opacity and bias. Target-specific ML applications further demonstrated practical impact; Ahmad and Raza (2024) showed CNN/RNN/GANs achieving over 95% accuracy in lung cancer QSAR and computer-aided design, validating the need for robust molecular representations addressed by our byte-level approach.

Public knowledge bases provide essential infrastructure for AI-driven discovery. Buniello et al. (2025) presented the Open Targets Platform integrating genetics, functional genomics, and pharmacology data with AI-powered literature summarization and target prioritization views featuring traffic-light scoring for clinical precedence and tractability. Zdrazil (2025) charted ChEMBL's development over 15 years into a FAIR bioactivity database supporting QSAR, ML benchmarking, and toxicology studies. Gingrich et al. (2025) presented canSAR 2024, a major cancer knowledgebase combining multi-omics and structural data, featuring an improved 3D ligandability classifier (positive-unlabeled learning) that doubles pocket detection to ~600,000 sites. Zhang et al. (2025) explained how bioinformatics databases, molecular docking, and multi-omics have become central to target identification and potency prediction for cancer and epidemic diseases such as COVID-19.

Articles by Jiang et al. (2025), Cao et al. (2025), Pitt et al. (2025), and Lee et al. (2025) explored cutting-edge architectures for molecular modeling. Jiang et al. reviewed transformer use in drug discovery, covering structure-aware models such as TopoFormer (with topological features), SMILES/reaction language models, and single-cell omics, but note high computational costs and interpretability issues. Cao et al. introduced InstructMol, a multimodal large language model aligning molecular graphs and sequences with natural language through instruction-tuning, achieving improved performance on property prediction, though limited by domain data scarcity and potential hallucination risks. Pitt et al. described human-in-the-loop workflows combining generative chemistry, QSAR predictors, and active learning. They found classical ML beats deep learning on typical project datasets, though GNNs helped with larger data when ADMET post-processing is applied. Lee et al. introduce GenMol, a fragment-based generalist model using masked discrete diusion on SAFE representations with fragment-level remasking and molecular context guidance, achieving the highest AUC (18.36) on the Practical Molecular Optimization (PMO) goal-directed benchmark and successfully improving binding scores in 26 of 30 docking-based lead optimization tasks.

Articles by Gangwal and Lavecchia (2025), Mondéjar-Parreño et al. (2025), An et al. (2025), and Li et al. (2025) addressed domain-specific challenges. Gangwal et al. reviewed AI in natural product drug discovery, surveying AI-enabled synthesis planning tools (Synthia, AiZynthFinder) and generative models for *de novo* NP-like molecule generation, with case studies demonstrating successes in identifying new antibiotics and kinase inhibitors while emphasizing limitations around NP structural complexity and synthetic inaccessibility. Mondéjar-Parreño et al. described new tools for antiarrhythmic development, including iPSC-derived cardiomyocytes, AI-multiomics biomarker discovery, and computational methods (QSAR, molecular dynamics) to revive a stalled pipeline. An et al. highlighted transformative technologies such as click chemistry, PROTACs, molecular glues, DNA-encoded libraries, and AlphaFold-enhanced CADD, emphasizing AI-multimodal data integration for optimal results. Li, Z. et al. proposed a framework combining spatial transcriptomics with U-Net segmentation and graph neural networks to jointly predict disease-specific biomarkers and classify tissue regions, achieving higher accuracy than baseline methods on brain and breast cancer datasets. Recent deep learning integrations with genomic profiling, such as Ahmad et al. (2025), enhance precision therapeutics through multimodal chemical-genomic models, complementary to our SMILES-focused potency prediction for downstream pipeline integration.

In these articles, one dominating theme can be observed: combining heterogeneous sources of information—molecular descriptors, graphs, sequences, omics data, and phenotype annotations—achieves a better level of predictive performance than any of the methods. As it is emphasized in previous articles (Rehman et al., 2025; Fu and Chen, 2025; Saini et al., 2025), AI should augment—not replace—human medicinal chemistry expertise. Future progress requires explainable models, federated learning for data privacy, and interpretable GNNs suitable for clinical translation. Previous articles (Jiang et al., 2025; Cao et al., 2025; Pitt et al., 2025) illustrate the progression of the traditional QSAR to the transformer works and generative models, which learn long-range dependencies and allow designs to be built in fragments. Target-specific studies show classical ML combined with deep learning, GNNs, and attention mechanisms can predict effectively across therapies—from Alzheimer's dual inhibitors to breast cancer and metabolic disease treatments.

Basic knowledge bases (Buniello et al., 2025; Zdrazil, 2025; Gingrich et al., 2025) and experimental platforms (Gangwal and Lavecchia, 2025; Mondéjar-Parreño et al., 2025; An et al., 2025) offer the platforms and validation structures. Key remaining challenges include data quality and bias, high computational demands, poor deep learning interpretability, regulatory hurdles for opaque models, and ensuring predictions remain reliable beyond well-characterized chemical spaces. The direction toward multimodal integration, pre-trained foundation models based on large chemical datasets, and the so-called virtual human simulators is the future of AI-enhanced pharmaceutical research, and the collaboration between computational scientists, medicinal chemists, and clinicians across the different fields will decide whether efforts are successful at making efforts to translate computational predictions to clinical outcomes.

Previous articles (Awomutia et al., 2025; Aman and Asnawi, 2024; Wang et al., 2021; Belaidi et al., 2024; Vignaux et al., 2023; Ajmani et al., 2013; Li et al., 2023; Bao et al., 2023; Wang et al., 2008; Gao et al., 2022) have worked using data in a similar SMILE format. Hence, we present the details of the models used in each article, their datasets, merits, and limitations in a tabular format in Table 1.

**Table 1:** Comparative analysis of models.

References	Models used	Dataset used	Performance	Merits	Limitations
Awomutia et al. (2025)	Light Gradient Boosting Machine (LightGBM) with molecular descriptors, Random Forest, Gradient Boosting, XGBoost, Bayesian Ridge, KNN	Environmental pollutants from 13 chemical categories	*R^2^* = 0.82 (training), *R^2^* = 0.59 (testing)	Interpretable feature importance; fast training	Lower test generalization; limited to PPARγ
Aman and Asnawi (2024)	Deep Neural Network (3 hidden layers, ReLU)	2,191 PPAR-binding compounds from ChEMBL	*R^2^* = 0.861 (training), *R^2^* = 0.655 (testing)	Captures non-linear relationships	Ignores molecular topology; moderate test performance
Wang et al. (2021)	QSAR using Ridge, Lasso, and SVR with applicability domain analysis	Large-scale PPARγ bioactivity datasets	Robust within defined applicability domains	Regulatory relevance; reliable predictions	Limited expressive power compared to deep learning
Belaidi et al. (2024)	LSTM, GRU, BiLSTM–BiGRU models on SMILES	15,872 SMILES-based compounds with pIC50 values	Best GRU: *R^2^* = 0.9036, MAE = 0.0213	High sequence-based prediction accuracy	Sensitive to SMILES format; low interpretability
Vignaux et al. (2023)	Machine learning classification models	Curated ChEMBL and BindingDB AChE datasets (7 species)	~81% accuracy for human AChE inhibition	Cross-species validation; highlights data curation	Manual curation effort; species-dependent performance
Ajmani et al. (2013)	QSAR using neural networks and statistical models	233 gamma-secretase inhibitors from ChEMBL	*r^2^* ≈ 0.70 (training)	Identifies key physicochemical descriptors	Small dataset limits generalization
Li et al. (2023)	Rule-based ML, classification trees, fragment-based design	AChE and BACE1 inhibitor datasets	Accuracy: 87% (AChE), 85% (BACE1); docking −12.31 to −21.20 kcal/mol	Dual-target inhibitor design; interpretable rules	Limited scalability; no deep learning
Bao et al. (2023)	Knowledge-BERT + CNN with attention mechanisms	Estrogen receptor α/β pIC50 datasets	MAE = 0.4983, *R^2^* = 0.9931 (test)	Very high accuracy via multi-modal learning	High complexity; risk of overfitting
Wang et al. (2008)	Bayesian-regularized neural networks with PCA	127 estrogen receptor α modulators	Correlation ≈ 0.91 (training), 0.90 (testing)	Strong performance on small datasets	Limited dataset size; no structural learning
Gao et al. (2022)	Gated Graph Neural Network (ABCD-GGNN)	ERα inhibitors and ADMET property datasets	*R^2^* = 0.7741; ADMET AUC up to 0.9714	Captures molecular topology and ADMET	Higher computational cost; slightly lower *R^2^*

## 3 Methods

**Figure 1:**
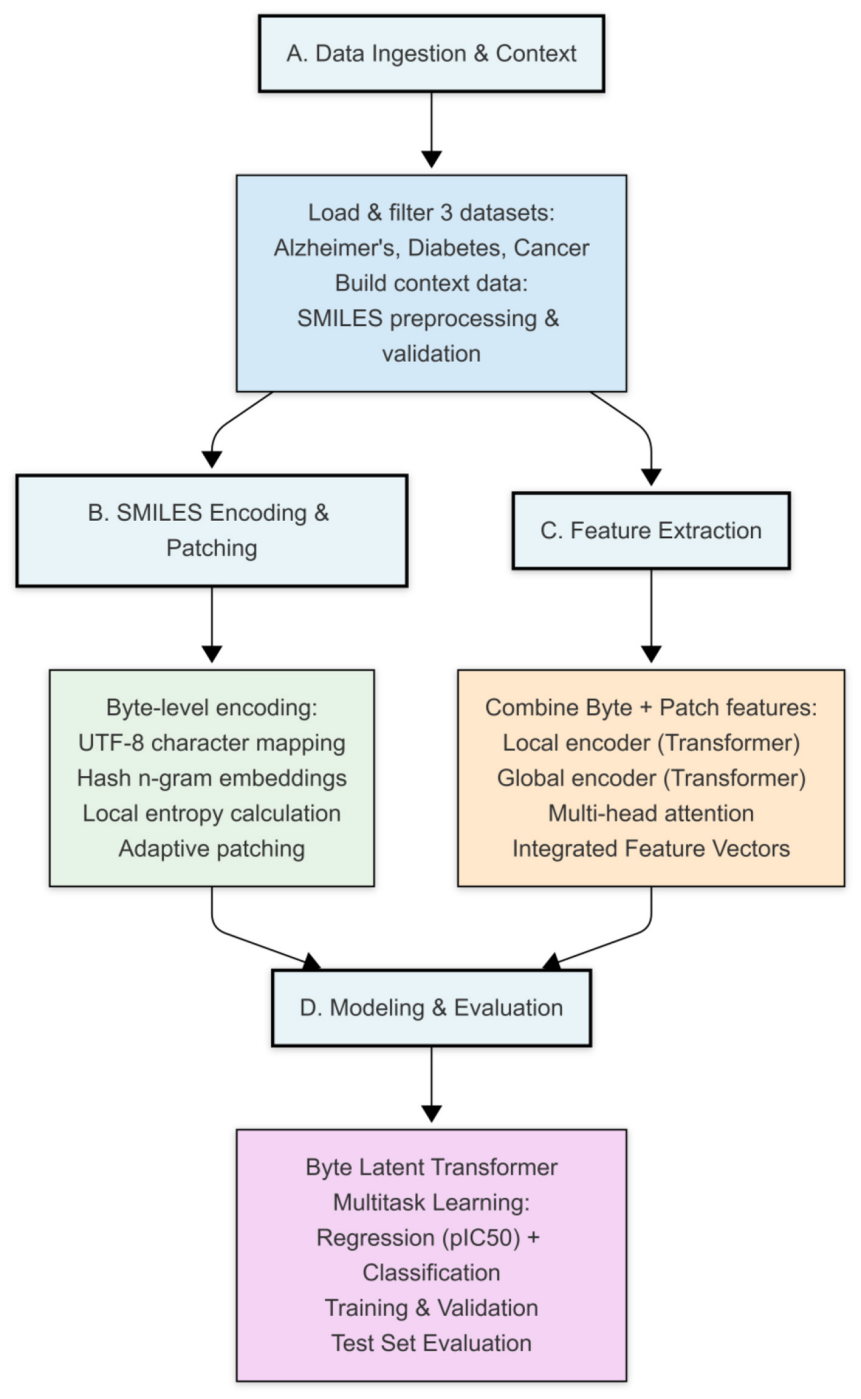
Workflow of the proposed potency prediction framework.

The proposed framework introduces a transformer-based molecular property prediction system that operates directly on byte-level SMILES representations. The architecture uses entropy-based adaptive patching to generate variable-length latent representations for multitask regression and classification, combined with hash n-gram embeddings. The overall flow of the process of the proposed molecular property predictive algorithm is shown in Figure 1.

### 3.1 Data acquisition and preprocessing

The framework goes on to start with bioactivity data collection and systematic preprocessing to determine the quality of data and the predictability of the models involved. Curated bioactivity datasets provide SMILES strings for chemical compounds alongside quantitative pIC50 potency measurements.

#### 3.1.1 Data cleaning and validation

Raw molecular data undergo a series of filtering procedures to remove inconsistencies and reach chemical validity. The preprocessing pipeline includes the removal of missing data, the verification of the SMILES syntax with the assistance of parsing and processing with the help of the RDKit, and the extraction of duplicates. For compounds with multiple bioactivity measurements, aggregation is performed by computing the median pIC50 value, represented as shown in Equation 1:


$$\begin{array}{l} pIC_{agg}\left(s\right)=median\left\{pIC50_{i}|SMILES_{i}=s\right\} \end{array}$$ (1)

where *s* denotes a unique SMILES string and *pIC*50_*i*_ represents individual measurements.

#### 3.1.2 Target standardization and binary labeling

Normalization of the continuity of pIC50 is done through z-score normalization to get a mean of zero and unit variance that easily optimizes gradients. The standardized target ŷ of a compound is determined by Equation 2:


$$\begin{array}{l} ŷ=\frac{\left(y-\mu \right)}{\sigma } \end{array}$$ (2)

where *y* represents the original pIC50 value, μ is the dataset mean, and σ denotes the standard deviation.

Binary activity labels for multitask learning are created by thresholding at each dataset's median pIC50 value. The binary classification target is defined as per Equation 3:


$$y_{cls}=\;\{\begin{array}{l} \;1\;if\;y_{original}\geq \tau \\ \;0\;otherwise \end{array}$$ (3)

Where τ represents the median pIC50 threshold separating active from inactive compounds.

#### 3.1.3 Density-based sample weighting

To correct for uneven sampling across the pIC50 distribution, we compute density-based sample weights via kernel density estimation. A Gaussian Kernel Density Estimation (KDE) is fitted to training targets, with inverse-density weighting upsampling sparse potency regions. The sample weight *w*_*i*_ for training instance *i* is given by Equation 4:


$$\begin{array}{l} w_{i}=\;\frac{1}{\rho \left(\hat{y_{i}}\right)+\epsilon } \end{array}$$ (4)

where $\rho \left(\hat{y_{i}}\right)$ denotes the kernel density estimate at the target value $\hat{y_{i}}$ and ϵ = 10^−8^ prevents division by zero. Weights are subsequently normalized such that their mean equals unity, as shown in Equation 5:


$$\begin{array}{l} w_{i}^{norm}=\frac{w_{i}}{\frac{1}{N}\sum_{j=1}^{N}w_{j}} \end{array}$$ (5)

Where *N* represents the total number of training samples.

#### 3.1.4 Data augmentation via smiles randomization

SMILES strings admit multiple canonical and non-canonical representations of the same molecular graph. Data augmentation generates k randomized SMILES variants per training molecule through atom reordering that preserves molecular identity, effectively expanding the training set k-fold while improving robustness to SMILES syntax variations.

### 3.2 Byte-level molecular encoding

Traditional molecular representation methods rely on predefined tokenization at the character or atom level. The suggested structure encodes a strategy to encode SMILES to a UTF-8 sequence of bytes such that it describes a universal vocabulary of size 256, and is not tokenized using domain-specific knowledge.

#### 3.2.1 Byte sequence representation

Each SMILES string *s* is encoded as a fixed-length byte sequence *b* = [*b*_1_, *b*_2_, …, *b*_*L*_], where *b*_*i*_∈{0, 1, …, 255} and *L* denotes the maximum sequence length. Short sequences that are less than L are right-padded with zero-bytes and long sequences are truncated. The encoding function is defined in Equation 6:


$$b=ByteEncode\left(s,L\right)=\{\begin{array}{l} \left[UTF-8\left(s\right)⊕0^{L-\left|s\right|}\right]\;if|s|<L\\ {\left[UTF-8s\right]}_{1:L}\;if\;|s|\geq L \end{array}$$ (6)

where ⊕ is concatenation, |·| is sequence length, and 0^*k*^ is a padding vector of *k* zeros.

#### 3.2.2 Hash n-gram feature embeddings

To encode the patterns of local substructure between the bytes, n-gram embedded by hash codes is included. With the *n*∈3, 4, 5, 6, sliding windows extracting teams of bytes of size n a, hashed to fixed-size embedding tables. Equation 7 is used to calculate the position i n-gram embedding as below.


$$\begin{array}{l} e_{ngram}^{\left(i\right)}=\underset{n\in N}{\sum }E_{n}\left[hash\left(b_{i:i+n}\right)\;mod\;V_{hash}\right] \end{array}$$ (7)

where *N* = 3, 4, 5, 6 denotes the set of n-gram sizes, $E_{n}\in R^{V_{hash}\times d}$ is the embedding matrix for n-grams of size *n*, *V*_*hash*_ is the hash table size, and *d* is the embedding dimension.

### 3.3 Entropy-based adaptive patching

Entropy-based dynamic patching—a key innovation—partitions byte sequences into variable-sized patches according to local predictability, allocating greater representational capacity to chemically complex regions while compressing routine substructures.

#### 3.3.1 Byte-level entropy estimation

An auxiliary transformer language model based on a small dataset is trained to improve the accuracy of the SMILES sequence of bytes in the next byte. The language model is a categorical distribution of the 256-byte vocabulary, at each location of a byte. Local entropy *H*_*i*_ is computed by use of Equation 8:


$$\begin{array}{l} H_{i}=-\overset{255}{\underset{v=0}{\sum }}p\left(b_{i+1}=v|b_{1:i}\right){\text{log}}_{2}p\left(b_{i+1}=v|b_{1:i}\right) \end{array}$$ (8)

where *p*(*b*_*i*+1_ = *v*|*b*_1:*i*_) is the conditional probability of observing byte value *v* at position *i*+1 given preceding context *b*_1:*i*_.

#### 3.3.2 Patch boundary determination

At places where the local entropy exceeds a given threshold τ_*H*_, patch boundaries are inserted, which suggests regions of great uncertainty or complexity in the structure. The patching algorithm is illustrated in Equation 9:


$$Patch\left(i\right)=\{\begin{array}{l} Patch\left(i-1\right)+1\;if\;H_{i}>\tau_{H}\\ Patch\left(i-1\right)\;otherwise \end{array}$$ (9)

in which *Patch*(*i*) is a patch identifier of the location of a byte at location *i*, and τ_H_ is a tuned entropy threshold, which is selected empirically. Other constraints limit the length of patches to a minimum and a maximum to eliminate degenerate segmentations.

### 3.4 Byte latent transformer architecture

**Figure 2:**
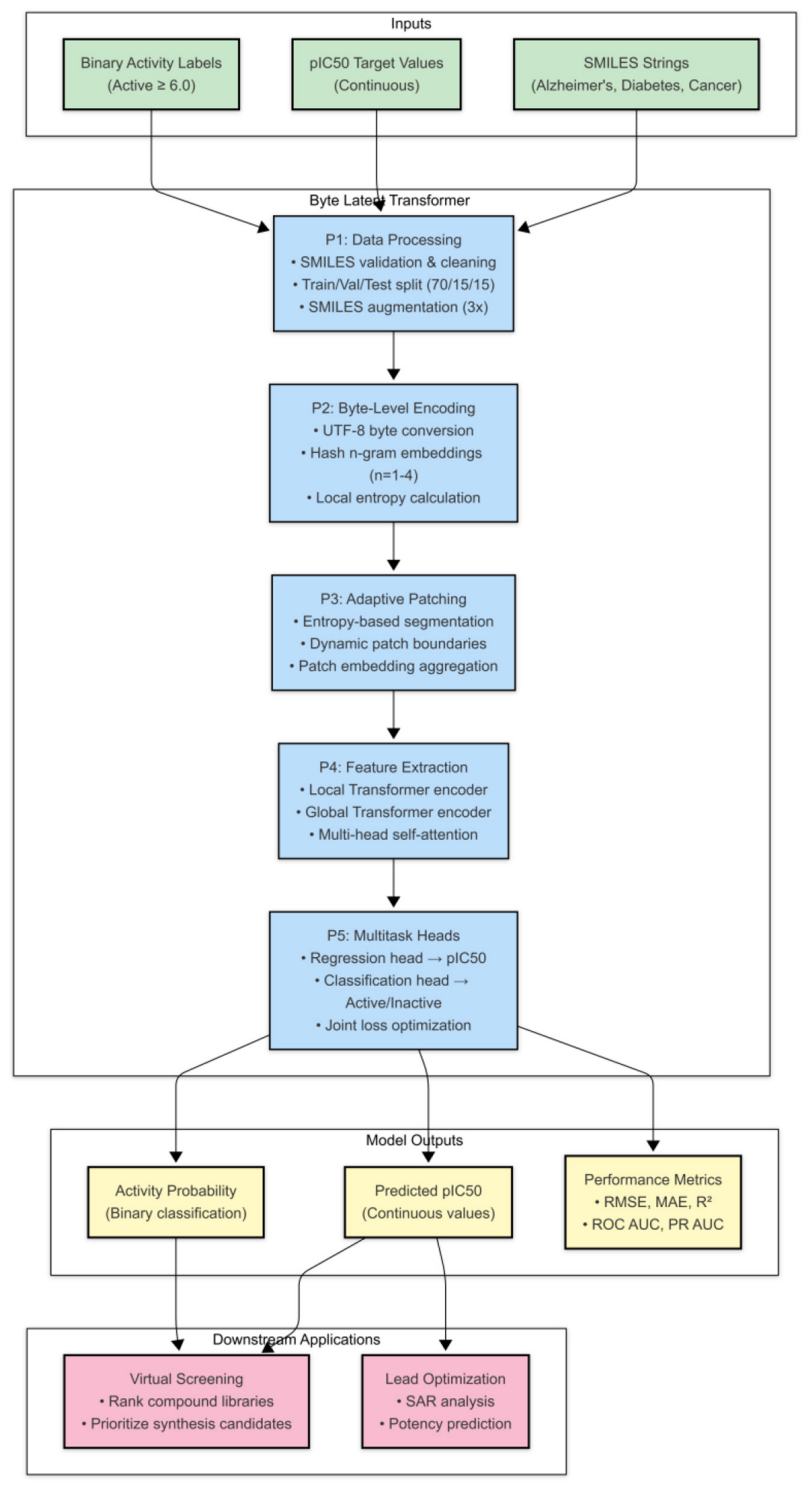
Architecture of the BLT model.

The core predictive model employs a hierarchical three-stage Byte Latent Transformer (BLT) (Pagnoni et al., 2025) architecture comprising a local encoder, global latent transformer, and local decoder. This hierarchical design processes long byte sequences efficiently by applying computationally intensive global self-attention only to compressed patch representations. The overall architecture is illustrated in Figure 2.

#### 3.4.1 Local encoder

The local encoder processes individual byte sequences to produce contextualized embeddings, which are then pooled into patch-level representations. Each byte *b*_*i*_ is first embedded into a continuous vector space via a learnable embedding matrix $E_{byte}\in R^{256\times d_{byte}}$. The vector is given by Equation 10:


$$\begin{array}{l} x_{i}=E_{byte}\left[b_{i}\right]+e_{ngram}^{\left(i\right)} \end{array}$$ (10)

where $e_{ngram}^{\left(i\right)}$ denotes the hash n-gram embedding from Equation 7.

The byte embeddings are processed through a multi-layer transformer encoder with windowed self-attention to capture local dependencies. The encoder transformation is given by Equation 11:


$$\begin{array}{l} H_{byte}=TransformerEncoder_{local}\left(X\right) \end{array}$$ (11)

Where *X* = [*x*_1_, *x*_2_, …, *x*_*L*_] and $H_{byte}\in R^{L\times d_{byte}}$ represents the contextualized byte-level hidden states.

Patch representations are derived by mean-pooling byte embeddings within each patch. For patch *p*, the aggregated representation *h*_*p*_ is computed as shown in Equation 12:


$$\begin{array}{l} h_{p}=\frac{1}{\left|B_{p}\right|}\underset{i\in B_{p}}{\sum }H_{byte}\left[i\right] \end{array}$$ (12)

where *B*_*p*_ = {*i*|*Patch*(*i*) = *p*} denotes the set of byte positions belonging to patch *p*.

The patch representations are subsequently projected to the global transformer dimension via a linear transformation specified in Equation 13:


$$\begin{array}{l} Z=LayerNorm\left(W_{proj}H_{patch}\right) \end{array}$$ (13)

where *H*_*patch*_ = [*h*_1_, *h*_2_, …*h*_*p*_], $W_{proj}\in R^{d_{patch}\times d_{byte}}$ is a learned projection matrix, and *P* denotes the total number of patches.

#### 3.4.2 Global latent transformer

The global latent transformer applies full self-attention across patch representations to capture long-range molecular structural dependencies. Positional embeddings are added to preserve patch ordering, as defined in Equation 14:


$$\begin{array}{l} Z^{\prime }=Z+E_{pos}\left[1:P\right] \end{array}$$ (14)

where $E_{pos}\in R^{P_{\max}\times d_{patch}}$ is a learned positional embedding matrix and *P*_max_ represents the maximum number of patches.

The enriched patch representations are processed through multiple transformer encoder layers, formalized in Equation 15:


$$\begin{array}{l} Z_{out}=TransformerEncoder_{global}\left(Z^{\prime }\right) \end{array}$$ (15)

Where $Z_{out}\in R^{P\times d_{patch}}$ contains the globally contextualized patch representations.

#### 3.4.3 Local decoder

The local decoder refines byte-level representations by attending to the global patch contexts via cross-attention mechanisms. Byte-level hidden states from the encoder serve as queries, while patch representations act as keys and values. The cross-attention operation is expressed in Equations 16–18:


$$\begin{array}{l} Q=W_{Q}H_{byte} \end{array}$$ (16)

$$\begin{array}{l} K=W_{k}Z_{out} \end{array}$$ (17)

$$\begin{array}{l} V=W_{V}Z_{out} \end{array}$$ (18)

where *W*_*Q*_, *W*_*K*_, *W*_*V*_ are learned projection matrices.

The attended representations are computed via scaled dot-product attention as shown in Equation 19:


$$\begin{array}{l} A=softmax\left(\frac{QK^{T}}{\sqrt{d_{byte}}}\right)V \end{array}$$ (19)

Residual connections and layer normalization stabilize training, formalized in Equation 20 using the value of A from Equation 19:


$$\begin{array}{l} H_{refined}=LayerNorm\left(H_{byte}+A\right) \end{array}$$ (20)

The refined byte representations are further processed through additional transformer decoder layers to produce the final byte-level outputs $H_{final}\in R^{L\times d_{byte}}.$

#### 3.4.4 Molecular representation pooling

To derive fixed-dimensional molecular representations suitable for property prediction, byte-level outputs are aggregated via masked mean pooling that excludes padding tokens. The pooled molecular representation *r* is computed according to Equation 21:


$$\begin{array}{l} r=\frac{\sum_{i=1}^{L}1\left[b_{i}\neq 0\right]\cdot H_{final}\left[i\right]}{\sum_{i=1}^{L}1\left[b_{i}\neq 0\right]} \end{array}$$ (21)

Where 1[·] denotes the indicator function.

### 3.5 Multitask learning framework

The framework employs multitask learning to simultaneously predict continuous potency values (regression) and binary activity labels (classification). Joint optimization leverages shared molecular representations with separate prediction heads for each task.

#### 3.5.1 Regression task head

Linear projection would be applied to one layer to predict continuous pIC50, whereby the pooled molecular representation would be projected to a scalar output as illustrated in Equation 22:


$${\hat{y}}_{reg}=w_{reg}^{T}r+b_{reg}$$ (22)

Where $w_{reg}\in R^{d_{byte}}$ and *b*_*reg*_∈*R* are learned parameters.

This is done by parallel linear projection followed by a sigmoid activation to classify the activities in a binary model with a loss function as indicated by Equation 23:


$$\begin{array}{l} L_{reg}=\frac{1}{N}\overset{N}{\underset{i=1}{\sum }}w_{i}^{norm}{\left({ŷ}_{reg}^{\left(i\right)}-y_{reg}^{\left(i\right)}\right)}^{2} \end{array}$$ (23)

where $w_{i}^{norm}$ is the normalized sample weight from Equation 5.

#### 3.5.2 Classification task head

Binary activity classification is done by a separate linear projection followed by sigmoid activation, as per Equation 24:


$${\hat{y}}_{cls}=\sigma \left(w_{cls}^{T}r+b_{cls}\right)$$ (24)

where σ(·) represents the sigmoid function.

The classification loss employs binary cross-entropy, formulated in Equation 25:


$$\begin{array}{l} L_{cls}=-\frac{1}{N}\overset{N}{\underset{i=1}{\sum }}\left[y_{cls}^{\left(i\right)}log{ŷ}_{cls}^{\left(i\right)}+\left(1-y_{cls}^{\left(i\right)}\right)log\left(1-{ŷ}_{cls}^{\left(i\right)}\right)\right] \end{array}$$ (25)

#### 3.5.3 Joint optimization objective

The total training loss combines regression and classification objectives via weighted summation, as shown in Equation 26:


$$\begin{array}{l} L_{total}=\lambda_{reg}L_{reg}+\lambda_{cls}L_{cls} \end{array}$$ (26)

where λ_*reg*_ and λ_*cls*_ are task-specific loss weights that balance the relative importance of each objective. Empirically, equal weighting (λ_*reg*_ = λ_*cls*_ = 1.0) is employed.

### 3.6 Training and optimization

Model parameters are optimized via the AdamW algorithm with learning rate warmup and cosine annealing schedules. The learning rate η_*t*_ at training step *t* is adjusted according to Equation 27:


$$\begin{array}{l} \;\eta_{t}\\ =\{\begin{array}{l} \eta_{\max}\cdot \frac{t}{T_{warmup}}\;if\;t\leq T_{warmup}\\ \eta_{\min}\text{​}+\text{​}\frac{1}{2}\left(\eta_{\max}\text{​}-\text{​}\eta_{\min}\right)\left(1\text{​}+\text{​}cos\left(\frac{t-T_{warmup}}{T_{\max}-T_{warmup}}\pi \right)\right)otherwise \end{array} \end{array}$$ (27)

Where η_max_ is the peak learning rate, η_min_ is the minimum learning rate, *T*_*warmup*_ denotes the warmup steps, and *T*_max_ represents the total training steps.

Gradient clipping with maximum norm 1.0 is applied to prevent training instability. The dataset is partitioned into training (70%), validation (15%), and test (15%) splits using stratified sampling based on binary activity labels to maintain class balance.

### 3.7 Algorithmic framework

The complete training procedure is outlined in Algorithm 1, which presents the pseudocode for the proposed molecular property prediction framework.

Algorithm 1 **Algorithm for byte latent transformer training.**

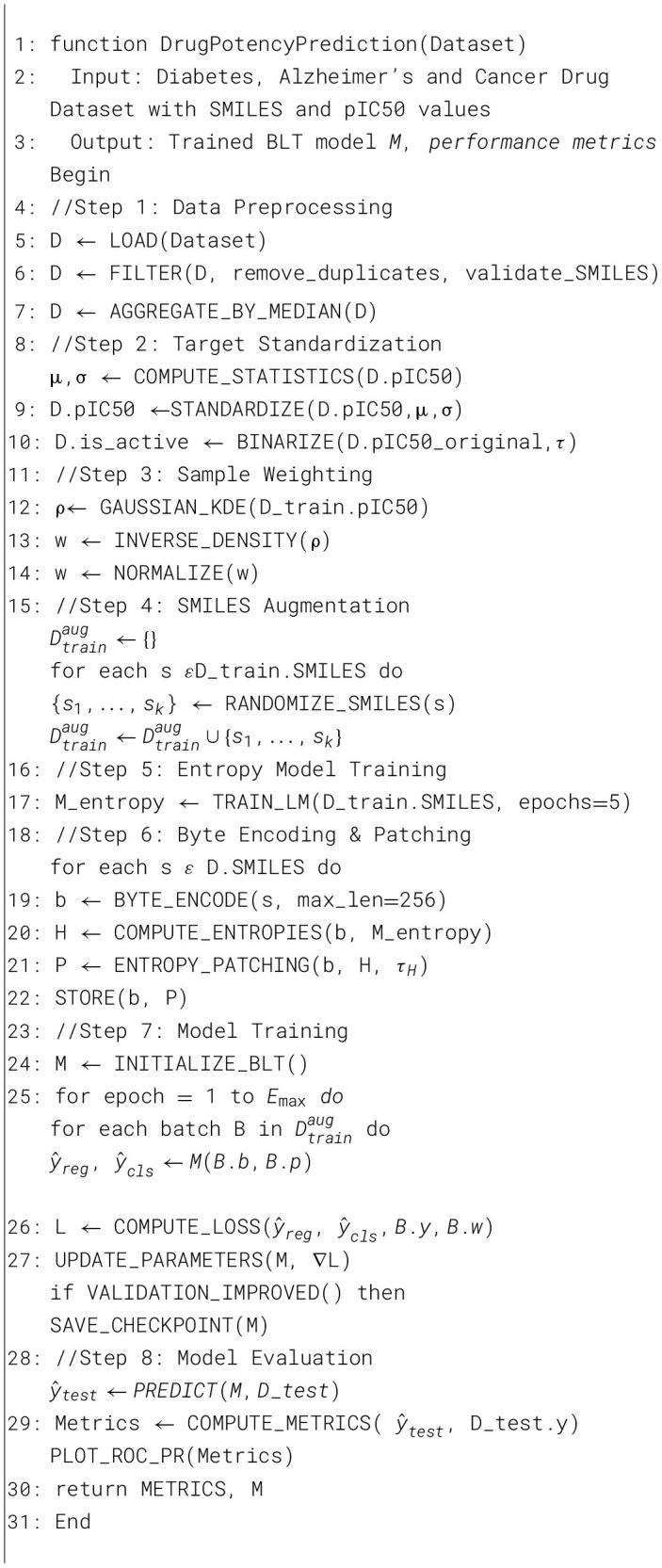

The framework combines data preprocessing, byte-level adaptive encoding, hierarchical transformers, and multitask learning for robust molecular property prediction from SMILES strings.

## 4 Results and discussion

This section presents the results and experimental setup for the proposed Byte Latent Transformer framework for target-drug pathogenicity classification. Model performance was evaluated on BindingDB datasets using standard regression and classification metrics, with comparisons to literature baselines.

### 4.1 Dataset description

The datasets used in this research were curated from BindingDB (Gao et al., 2022), a public database containing experimentally measured binding affinities for small molecules against protein targets relevant to drug discovery. Three disease-specific datasets were constructed by querying BindingDB for bioactivity data against therapeutically relevant protein targets: BACE1 (β-secretase 1) and AChE (acetylcholinesterase) for Alzheimer's disease, PPAR-γ (peroxisome proliferator-activated receptor gamma) and PPAR-α for diabetes, and ERα (estrogen receptor alpha) for cancer. Each dataset comprises SMILES (Simplified Molecular Input Line Entry System) representations of drug-like small molecules paired with experimentally measured pIC50 values, where pIC50 = –log10(IC50) quantifies compound potency against the respective biological targets. Table 2 summarizes the statistical characteristics of each dataset.

**Table 2:** Dataset statistics for three disease domains.

Dataset	Total compounds	Mean pIC50 ±Std	Min pIC50	Max pIC50	Active (≥6.0pIC)	Inactive (< 6.0pIC)	Active %
Alzheimer's	2,270	5.846 ± 1.348	2.781	9.523	832	1,438	36.65
Diabetes	3,058	6.451 ± 1.114	3.000	9.886	1,863	1,195	60.92
Cancer	3,255	7.366 ± 1.591	2.456	11.000	2,487	768	76.41

Readings on binary activity were determined at a threshold pIC50 ≥ 6.0, equivalent to IC50 ≤ 1 μM, a typical cutoff point of hits with a drug of biological interest. The Alzheimer and diabetes data are equal concerning the ratio of active to inactive (0.579 and 1.559, respectively), as compared to the cancer data that is imbalanced between the classes (ratio 3.238) and favorable to the active compounds, which is addressed by correctly weighting the losses during the training. All datasets underwent SMILES validation, standardization, invalid structure removal, and stratified splitting (70% train, 15% validation, and 15% test) to preserve class balance. To perform deep learning and PyTorch simulation and analysis, scikit-learn to assess metrics, pandas to preprocess, NumPy to perform numerical operations, and RDKit to SMILES validate, Python and PyTorch were used.

### 4.2 Implementation details

The model that was used in this experiment was coded in Python as a PyTorch-based deep learning pipeline and scikit-learn-based evaluation performance. Training used NVIDIA Tesla T4 GPUs with mixed-precision computation to minimize memory usage and speed up convergence. The byte-level language model for entropy estimation was pre-trained for 5 epochs on the training set SMILES strings before being frozen for patch boundary determination.

Hyperparameters for the Byte Latent Transformer were configured as follows: byte embedding dimension *d*_*byte*_ = 128, patch embedding dimension *d*_*patch*_ = 256, local encoder layers = 4, global transformer layers = 6, attention heads = 8, and maximum sequence length = 256 bytes. Training employed the AdamW optimizer with initial learning rate 1 × 10^−3^, linear warmup for 500 steps, and cosine annealing decay. SMILES augmentation generated three randomized variants per training molecule, expanding the effective training set by 3-fold.

### 4.3 Performance metrics

The performance of the proposed model is evaluated using two complementary sets of metrics: regression metrics for continuous pIC50 prediction and classification metrics derived from binarized activity labels.

#### 4.3.1 Regression metrics

For the primary task of continuous pIC50 prediction, standard regression evaluation metrics are employed. The Root Mean Squared Error (RMSE) quantifies the average magnitude of prediction errors in pIC50 units, defined as shown in Equation 28:


$$\begin{array}{c} RMSE=\sqrt{\frac{1}{N}\overset{N}{\underset{i=1}{\sum }}{\left(\hat{y_{i}}-y_{i}\right)}^{2}} \end{array}$$ (28)

Where *N* denotes the number of test samples, $\hat{y_{i}}$ represents the predicted pIC50, and *y*_*i*_ is the true pIC50 value.

The MAE is more resistant to outliers and provides a superior measure of the average absolute deviation, as shown by Equation 29:


$$MAE=\frac{1}{N}\sum_{i=1}^{N}\left|\hat{y_{i}}-y_{i}\right|$$ (29)

The Coefficient of Determination (*R*^2^ Score) is the ratio of the change in the actual values that are explained by the model predictions, and it is represented by Equation 30:


$$R^{2}=1-\frac{\sum_{i=1}^{N}{\left(y_{i}-\hat{y_{i}}\right)}^{2}}{\sum_{i=1}^{N}{\left(y_{i}-\bar{y}\right)}^{2}}$$ (30)

Where ȳ represents an average of true pIC50. *R*^2^ can range between negative infinity and 1, where 1 is a perfect predictor, and closer to 0 indicates predictive performance as close to a perfect predictor as possible.

In addition, the linearity desired between predictions and true values that are calculated according to the equation is quantified via the Pearson correlation coefficient as per Equation 31:


$$r=\frac{\sum_{i=1}^{N}(y_{i}-\bar{y})\left(\hat{y_{i}}-\bar{\hat{y}}\right)}{\sqrt{\sum_{i=1}^{N}{\left(y_{i}-\bar{y}\right)}^{2}}\sqrt{\sum_{i=1}^{N}{\left(\hat{y_{i}}-\bar{\hat{y}}\right)}^{2}}}$$ (31)

The Spearman rank correlation coefficient is a non-parametric statistic that is not sensitive to non-linear monotonic correlation or outliers.

#### 4.3.2 Classification metrics

The binarization is done continuously using the median pIC50 dataset threshold τ of each dataset to enable it to be compared to classification-based approaches and to ascertain the discrimination capability of the model between active and inactive compounds. Binary classification is as per Equation 32:


$${\hat{y}}_{binary}=\{\begin{array}{l} 1\;if\;{\hat{y}}_{reg}\geq \tau \\ 0\;otherwise \end{array}$$ (32)

The final confusion matrix is utilized to compute Standard classification measures of accuracy, precision, recall (True Positive Rate), and F1-score. ROC AUC quantifies discrimination across all classification thresholds, plotting true positive rate vs. false positive rate. Precision-Recall Area Under the Curve (PR AUC) is particularly informative when the datasets are not balanced and is focused on a trade-off between precision and recall.

These families of complementary metrics provide the complete assessment of the accuracy of regression and binary classification.

### 4.4 Regression performance analysis

The general regression performance measures of the proposed Byte Latent Transformer in each of the three disease domains are revealed in Table 3. These regression performance results were obtained from the holdout external test set (15%).

**Table 3:** Regression performance metrics.

Dataset	MSE	RMSE	MAE	*R^2^* score	Pearson coeff.	Spearman coeff.
Alzheimer's	0.0382	0.1954	0.1541	0.9807	0.9905	0.9800
Diabetes	0.0445	0.2109	0.1683	0.9569	0.9795	0.9736
Cancer	0.0555	0.2355	0.1679	0.9791	0.9901	0.9888

The designed model is very predictive, and it is seen to be accurate in all the datasets that were used in the study. RMSE, MAE, and R2 of the model on the diabetes dataset stand at 0.2109, 0.1683, and 0.9569, indicating a high degree of fidelity of pIC50 prediction. Pearson correlation coefficient of 0.9795 and Spearman correlation of 0.9736 guarantee that both linear and monotonic correlation exist between the predicted and the true values, respectively.

The model gave similar values on the Alzheimer dataset with RMSE of 0.1954, MAE of 0.1541, and R2 of 0.9807. Cancer dataset demonstrates a similar performance with RMSE equal to 0.2355, MAE equal to 0.1679, and R2 equal to 0.9791. High *R*^2^ values across diseases demonstrate that byte-level SMILES encoding with entropy-based adaptive patching captures most pIC50 variance effectively.

**Figure 3:**
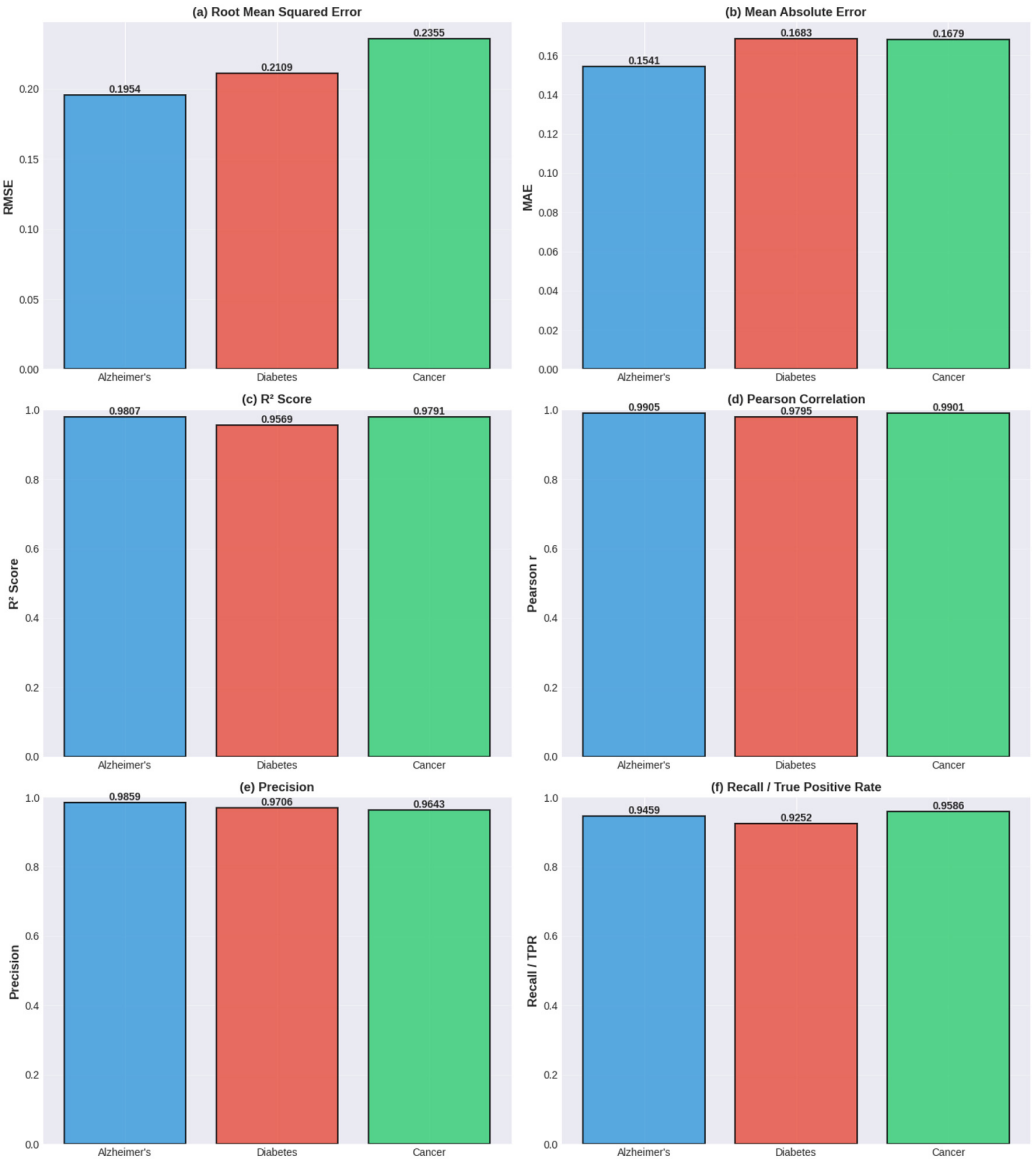
Performance metrics comparison across diseases (Alzheimer's, Diabetes, and Cancer) **(a)** RMSE, **(b)** MAE, **(c)** *R*^2^, **(d)** Pearson Correlation, **(e)** Precision, and **(f)** Recall.

The visual comparison of the three essential regression and classification measures in the three domains of diseases is presented in Figure 3.

Figure 3 shows consistent performance across diseases. Alzheimer's yields the lowest RMSE (Figure 3a) and highest *R*^2^ (Figure 3c), while MAE remains low across datasets (Figure 3b). High Pearson correlations (Figure 3d) confirm strong linear agreement with experimental values.

**Figure 4:**
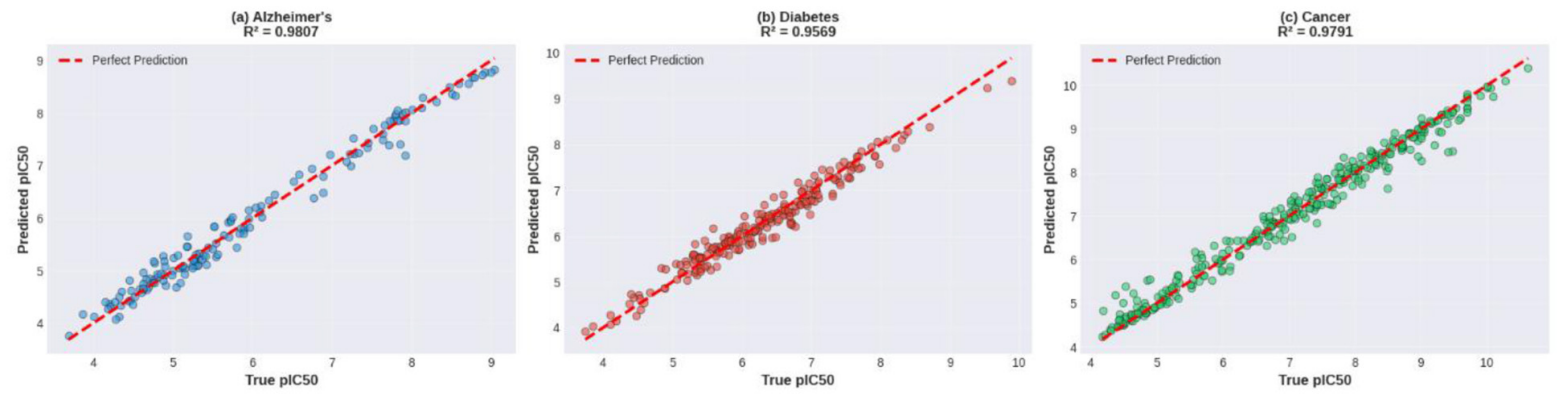
Predicted vs. true pIC50 scatter plots for **(a)** Alzheimer's, **(b)** Diabetes, and **(c)** Cancer.

The scatter plots of predicted and true values of pIC50 in each disease domain mentioned in Figure 4 will give visual evidence of the quality of prediction.

Figure 4 indicates that all three of the datasets have a tight distribution around the optimal 45-degree line (indicating perfect predictions). The Alzheimer data (Figure 4a) show a very close correlation with R2 = 0.9807, as shown by the fact that there is very little deviation from the reference line. In the case of the diabetes dataset (Figure 4b), the model exhibits low systematic bias with the range of potency. The similar results can be seen in the cancer dataset (Figure 4c). Residual plots show no strong heteroscedasticity—prediction reliability remains consistent across the full potency range.

### 4.5 Classification performance analysis

Although the main task is a continuous pIC50 regression, the ability of the model to classify binary activities gives complementary useful information. Table 4 is a summary of classification metrics based on median-thresholded prediction. These classification metrics are derived from a subset of the test dataset used for regression metrics by using dataset-specific median pIC50 thresholding. This data-adaptive binarization was done to balance active/inactive data point classes per disease domain.

**Table 4:** Classification performance metrics across disease domains.

Disease	Accuracy	Precision	Recall	F1 score	ROC-AUC	PR-AUC	FPR
Alzheimer's	0.9660	0.9859	0.9459	0.9655	0.9954	0.9957	0.0137
Diabetes	0.9484	0.9706	0.9252	0.9474	0.9878	0.9885	0.0283
Cancer	0.9614	0.9643	0.9586	0.9614	0.9957	0.9960	0.0357

The model proposed performs well on all the datasets in terms of classification. With the diabetes dataset, accuracy is at 94.84% with a precision of 0.9706 and a recall of 0.9252, which gives a F1-Score of 0.9474. The ROC AUC of 0.9878 and PR AUC of 0.9885 are good signs of excellent discriminative ability among active and inactive compounds.

The Alzheimer dataset has better classification indicators with an accuracy of 96.60%, ROC AUC of 0.9954, and PR AUC of 0.9957. In the case of the cancer data, the model is achieved with 96.14% accuracy, ROC AUC of 0.9957, and PR AUC of 0.9960. The high ROC AUC values, which are maintained in all diseases, indicate strong ranking capabilities, which are required for virtual screening use.

False positive rates remain below 5% across datasets, with recall exceeding 94%, yielding balanced F1-scores that indicate no systematic bias toward conservative or aggressive classification.

**Figure 5:**
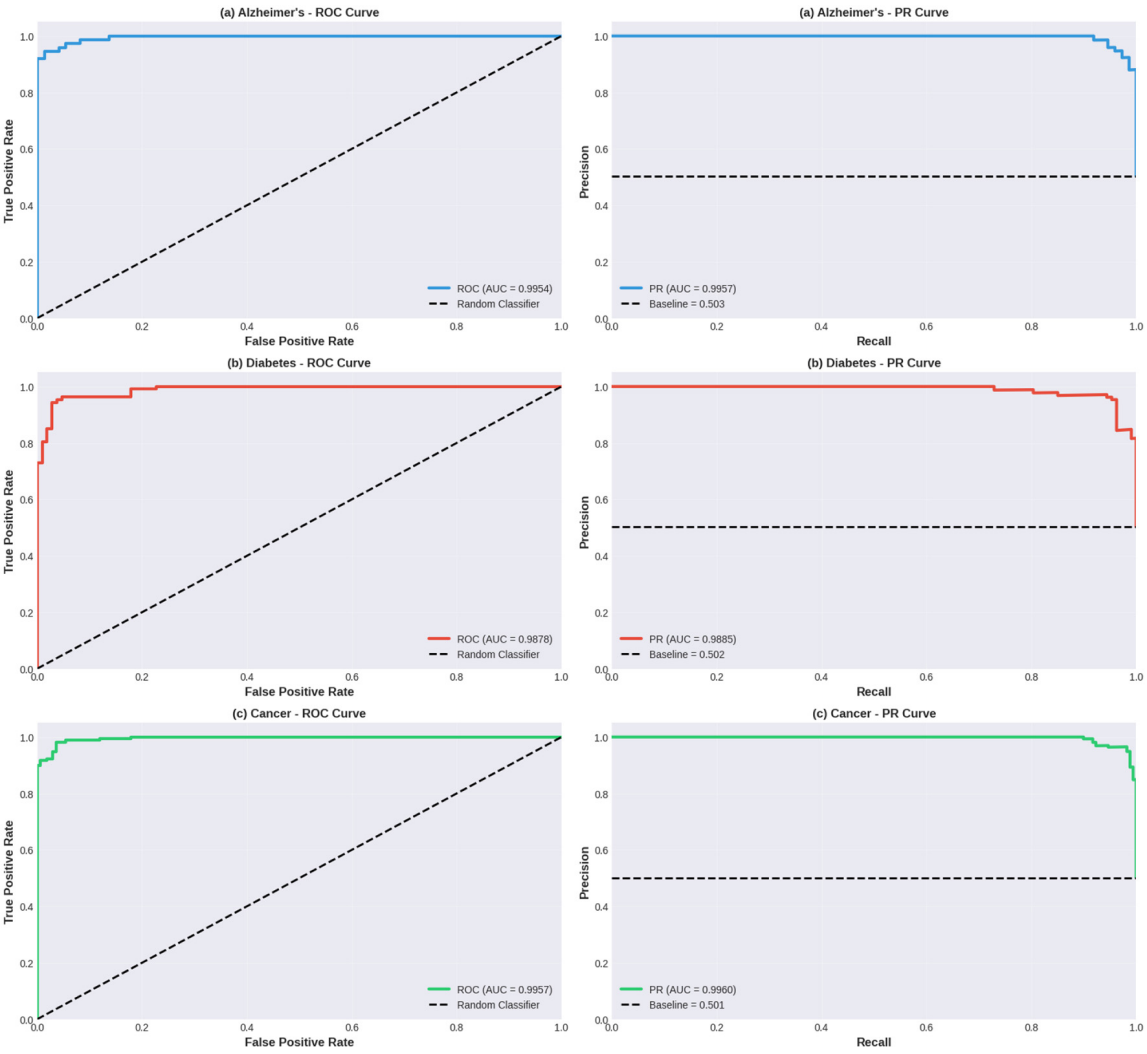
ROC and precision-recall curves for binary classification—**(a)** Alzheimer's, **(b)** Diabetes, and **(c)** Cancer.

The ROC and precision-recall curves of the three disease datasets are plotted adjacent to one another, in Figure 5, and indicate the discriminative capability of the model at different decision thresholds and the precision-recall trade-off.

It is seen that the ROC curves in Figure 5 have better performance than the random grouping (diagonal baseline) of all datasets. The dataset with the highest ROC AUC of 0.9957 is the cancer dataset (Figure 5c), whose curve has almost touched the desired optimal position of the top-left corner. Alzheimer's (Figure 5a) and diabetes (Figure 5b) datasets exhibit similar values of AUC of less than the values of 0.9954 and 0.9878, respectively, all suggest high discriminative capacity. The steep initial rise in all ROC curves suggests that the model can achieve high True Positive Rates while maintaining low False Positive Rates, a desirable characteristic for compound prioritization workflows.

The precision-recall curves substantially outperform the baseline random classifier. The datasets achieved high PR AUC, indicating that high precision can be maintained even at elevated recall levels. Precision-recall curves maintain high precision across recall values, confirming effective active compound retrieval with minimal false positives—critical for resource-constrained hit validation.

Table 5 has in-depth statistics of the confusion matrix, a table that measures the correct classifications and error modes.

**Table 5:** Confusion matrix values for binary classification.

Disease	True positives	True negatives	False positives	False negatives
Alzheimer's	70	72	1	4
Diabetes	99	103	3	8
Cancer	162	162	6	7

As can be seen, Table 5 indicates that the model has high counts of True Positives and True Negatives in all datasets, having a comparatively small number of False Positives and False Negatives. For the diabetes dataset, the model correctly identifies 162 active and 162 inactive compounds, misclassifying only three inactives as active and missing eight actives. Similar performance holds for Alzheimer's and cancer datasets.

**Figure 6:**
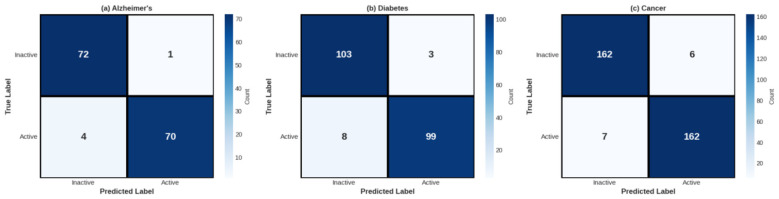
Confusion matrices: **(a)** Alzheimer's, **(b)** Diabetes, and **(c)** Cancer.

These confusion matrices are visualized in Figure 6 in the form of heatmaps, which allows intuitively interpolating the performance of classification.

Figure 6 reveals that the confusion matrix heatmaps are characterized by a high level of diagonal dominance, which suggests that most of the predictions are made to be correct. Low off-diagonal values in confusion matrices confirm minimal misclassification. This generalization across chemical diversity reflects robust discriminative feature learning.

### 4.6 Error analysis and residual distributions

**Figure 7:**
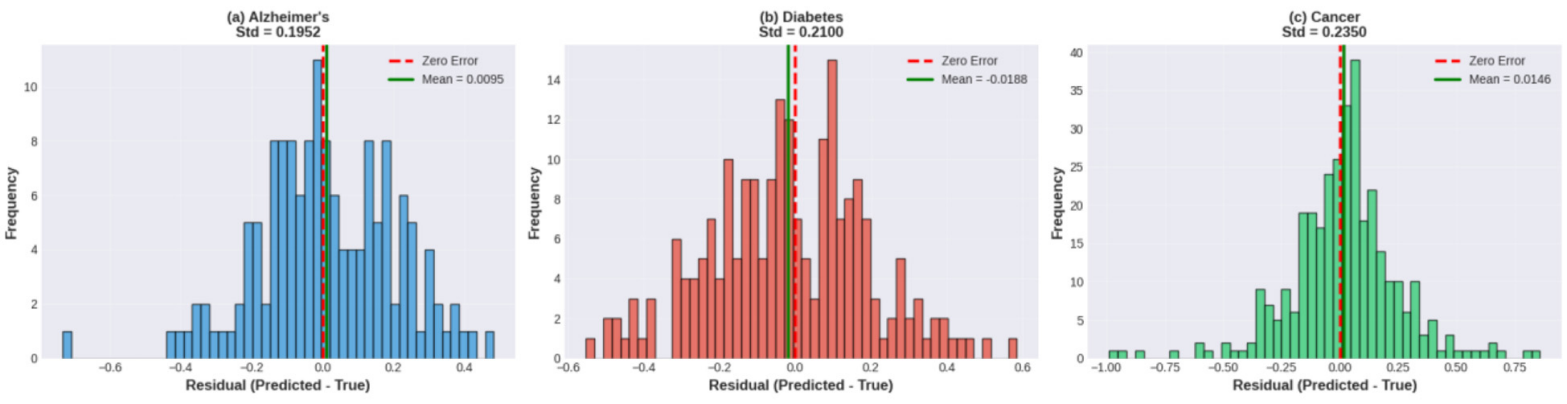
Residual distribution analysis: **(a)** Alzheimer's, **(b)** Diabetes, and **(c)** Cancer.

Understanding prediction error distributions reveals model reliability and identifies potential failure patterns. Figure 7 shows the histograms of residuals of all three datasets of diseases.

Figure 7 shows that the residual distributions are approximately Gaussian in shape, and we can say that there is no systematic prediction bias. For the diabetes dataset (Figure 7b), the mean residual is−0.0188 with standard deviation 0.2100, suggesting unbiased predictions with controlled variance. The Alzheimer's (Figure 7a) and cancer (Figure 7c) datasets show similar patterns with mean residuals of 0.0095 and−0.0146 and standard deviations of 0.1952 and 0.2350, respectively.

Tight residual distributions show few large errors, with approximate symmetry indicating no systematic over- or under-prediction bias. Tail deviations reflect occasional structurally unusual test compounds.

**Figure 8:**
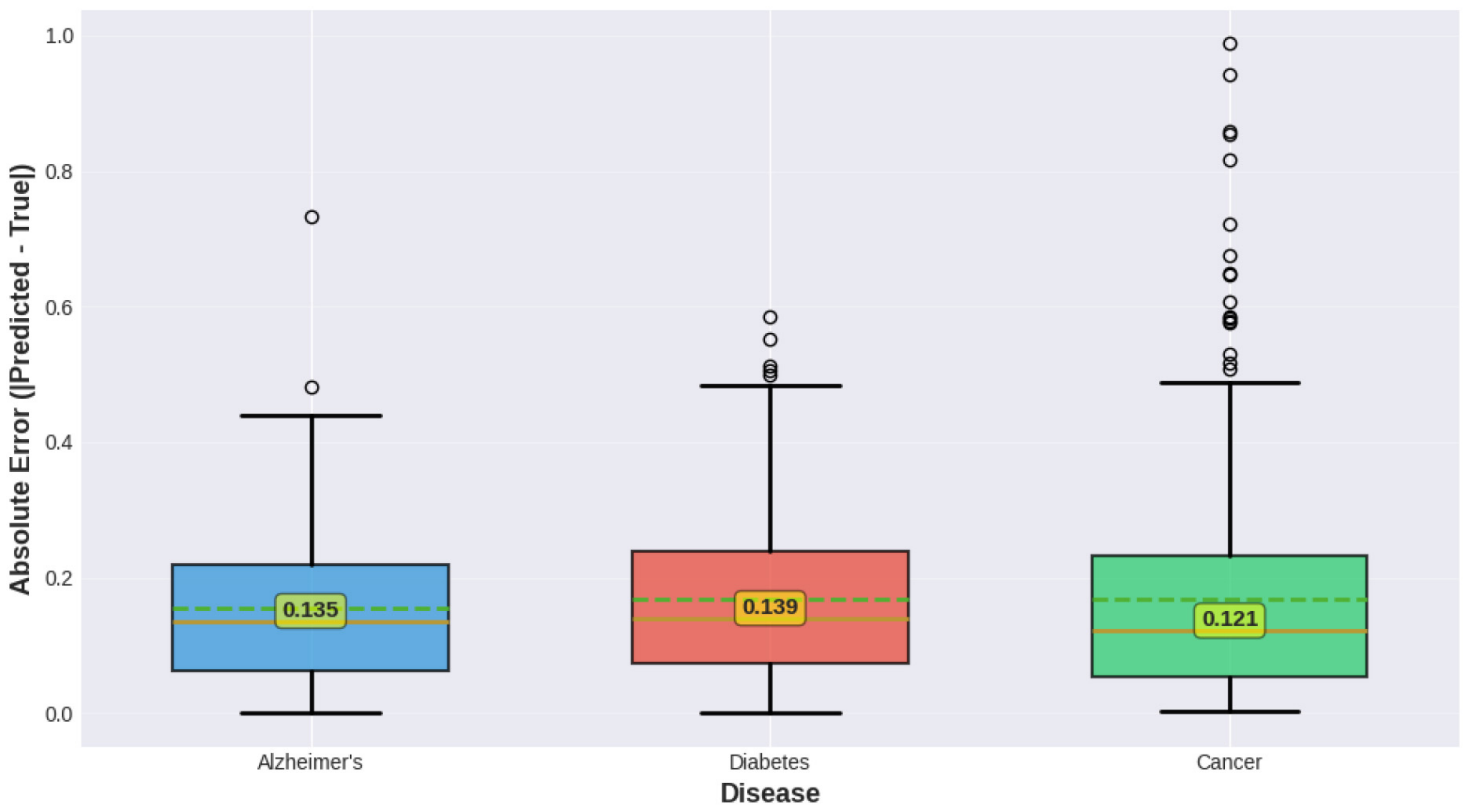
Absolute error distribution across diseases.

Figure 8 provides a comparative view of absolute error distributions across diseases using box plots.

The box plots of Figure 8 indicate similar distributions of error in the three domains of the disease. The median errors of absolute errors lie between 0.121 and 0.139 pIC50 units, which is reasonably acceptable for initial compound prioritization. Compact interquartile ranges confirm consistently high prediction quality across most test compounds.

Outliers represent challenging molecules, but their low frequency indicates that these are rare exceptions rather than systematic weaknesses. The fact that the lengths of the whiskers are relatively symmetric shows that both over-predictions and under-predictions are equally uniformly represented.

### 4.7 Comparative analysis with existing methods

We compared BLT on Random Splitting's performance against literature models trained on identical BindingDB datasets to enable direct evaluation. This comparative analysis is provided in Table 6 in several different approaches considered on the same regression and classification tasks to predict molecular bioactivity.

**Table 6:** Comparison of proposed method with existing approaches using random splitting.

Disease	Model	*R^2^* score	MAE
Diabetes (PPAR-Gamma and Alpha)	**Proposed**	**0.956923**	**0.168325**
	SVR (Wang et al., 2021)	0.67283	0.706332
	LightGBM (Awomutia et al., 2025)	0.5999	0.5823
	MLP (Aman and Asnawi, 2024)	0.655	0.436
Alzheimer's (BACE1 and AChE)	**Proposed**	**0.98069**	**0.154134**
	LSTM (Belaidi et al., 2024)	0.9031	0.0263
	GRU (Belaidi et al., 2024)	0.9073	0.0202
	kNNR (Vignaux et al., 2023)	0.76	0.53
Cancer (ERα)	**Proposed**	**0.979071**	**0.167932**
	Knowledge-BERT-1D-ECA-CNN (Li et al., 2023)	0.7791	0.4983
	Attentive FP (Li et al., 2023)	0.7521	0.5112
	ABCD-GGNN (Gao et al., 2022)	0.7741	-

Bold indicates values corresponding to BLT Based Architecture proposed in this work.

### 4.8 Ablation study, scaffold splitting, and further baseline comparisons

To stringently test the generalization property of the BLT operating on simple random splits, which may leak information via structural similarity, we conducted scaffold-based splitting. This divides test sets according to molecular scaffolds (Bemis–Murcko frameworks) (Bemis and Murcko, 1996), so that no test compounds have core structures in common with training data, so as to simulate prospective screening in the real world, in which novel chemotypes must be predicted.

Table 7 is an Ablation Study, which compares the system with and without the Entropy-Based Adaptive Patching in the same scaffold splits. Entropy mode is observed to yield good results as compared to fixed patching in all diseases. This confirms our hypothesis: the dynamically distributed representational capacity to chemically complex regions enhances extrapolation to invisible scaffolds. In addition, we also observe that the *R*^2^ values of applying the Scaffold Splitting are a little smaller than those obtained with using Random Splitting in Table 6, although even after applying the Scaffold Splitting, the model still records rather high values.

**Table 7:** BLT tested with and without entropy mode under scaffold splitting.

Dataset	Patching mode	MAE	RMSE	*R^2^* score
Cancer	Entropy based	0.2447	0.3381	0.9555
Diabetes	Entropy based	0.2135	0.2874	0.9156
Alzheimer's	Entropy based	0.1783	0.2289	0.9712
Cancer	Fixed	0.2847	0.3667	0.9284
Diabetes	Fixed	0.2442	0.3047	0.8884
Alzheimer's	Fixed	0.2389	0.3059	0.9211

**Figure 9:**
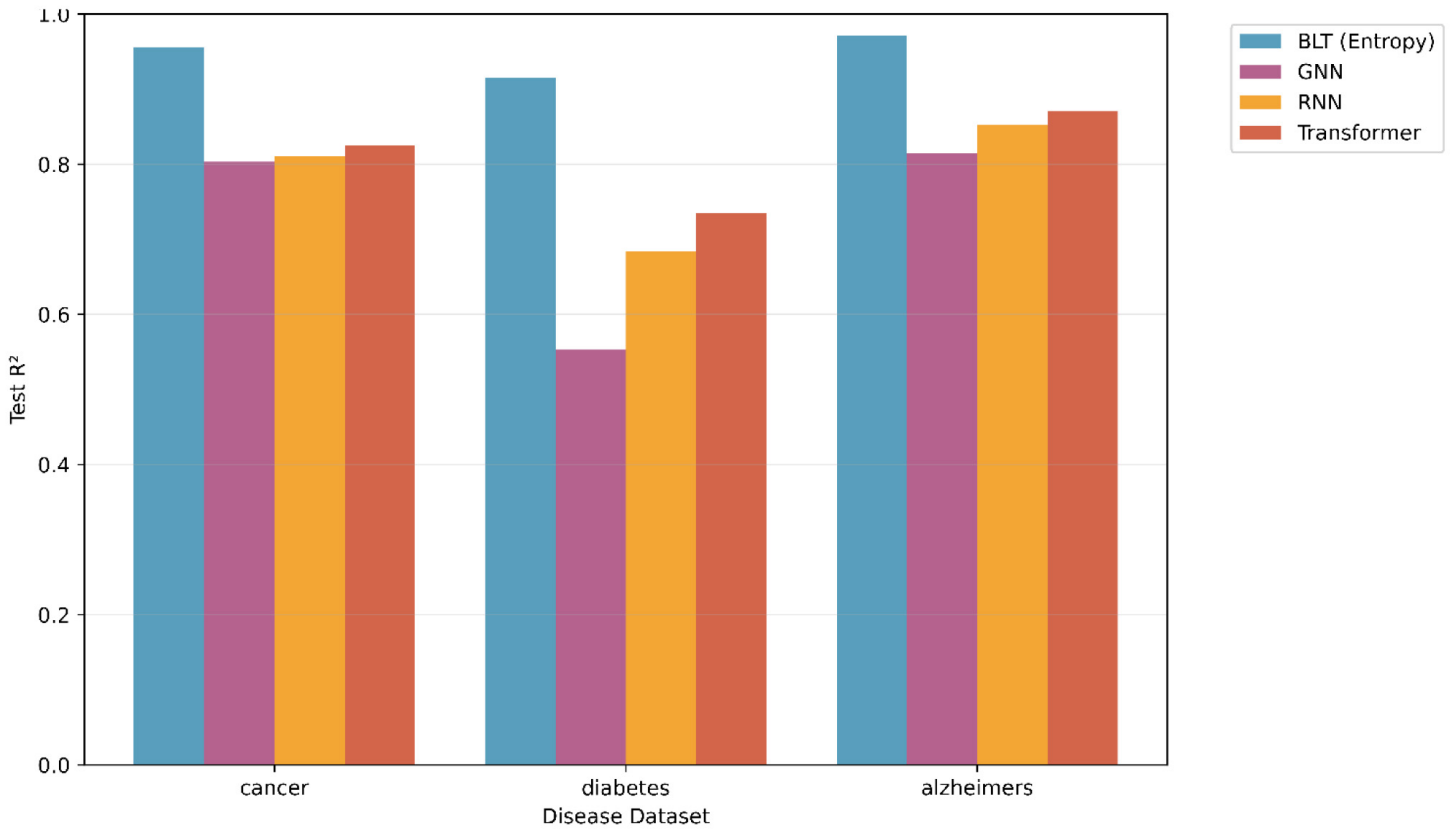
*R*^2^ Score comparison for BLT and baseline models locally trained across the same datasets using scaffold-based splitting.

Table 8 and Figure 9 include head-to-head comparisons with three locally trained baselines: Graph Neural Networks (GNNs), Recurrent Neural Networks (RNNs), and a SMILE Transformer (Honda et al., 2019) trained on the identical scaffold-split datasets as BLT. BLT shows significant improvement over all baselines, with a significant increase in *R*^2^ scores in all the three diseases. This is also in addition to the fact that BLT has a hierarchical modeling of the scaffold-invariant chemical patterns that are not captured by the graph/sequence models.

**Table 8:** Comparison of proposed method with locally trained baseline models using scaffold-based splitting.

Disease	Model	*R^2^* score	MAE
Diabetes (PPAR-Gamma and Alpha)	**Proposed**	**0.9156**	**0.2135**
	SMILE transformer	0.7350	0.3773
	RNN	0.6834	0.4154
	GNN	0.5529	0.4960
Alzheimer's (BACE1 and AChE)	**Proposed**	**0.9712**	**0.1783**
	SMILE transformer	0.8704	0.3762
	RNN	0.8530	0.3797
	GNN	0.8150	0.4453
Cancer (ERα)	**Proposed**	**0.9555**	**0.2447**
	SMILE transformer	0.8250	0.5015
	RNN	0.8114	0.5311
	GNN	0.8035	0.5383

Bold indicates values corresponding to BLT Based Architecture proposed in this work.

## 5 Conclusion

This article has introduced a Byte Latent Transformer architecture to predict molecular properties in three disease-specific contexts, namely Alzheimer's, diabetes, and cancer. The given approach uses encoded SMILES at the level of bytes along with entropy-based adaptive patching and hash n-gram encodings to encode not only local patterns of the substructure but also global molecular syntax without any hand-crafted features. The model was worked and tested on curated bioactivity datasets, indicating the best performance on Alzheimer BACE1 +AChE dataset, where the *R*^2^ scores were high with 0.98069 and RMSE was low at 0.195404 pIC50 units. The model matches or exceeds graph neural networks and traditional ML baselines across regression metrics (RMSE, MAE, and *R*^2^) and classification metrics (ROC AUC, and PR AUC). The multitask learning architecture supports binary activity classification and continuous prediction of potency at the same time, allowing two capabilities that will be useful in drug discovery processes.

Future work can extend the framework to incorporate 3D conformations via equivariant transformers and geometric deep learning for structure-based potency modeling. Transfer learning from large pre-trained chemical foundation models will address data-scarce targets, while multi-task expansion to ADMET screening with frameworks like DrLungker (Ahmad and Raza, 2026) can enable an end-to-end hit-to-lead pipeline. Bayesian uncertainty quantification will provide confidence intervals for experimental prioritization, and the entropy-based patching mechanism can be enhanced with learnable thresholds and attention-guided segmentation. Finally, integration into production-scale virtual screening pipelines against novel targets will validate practical utility for hit identification and structure-guided lead optimization in real drug discovery campaigns.

## Data Availability

The original contributions presented in the study are included in the article/supplementary material, further inquiries can be directed to the corresponding author.
